# Disrupted White Matter Microstructure of the Cerebellar Peduncles in Scholastic Athletes After Concussion

**DOI:** 10.3389/fneur.2019.00518

**Published:** 2019-05-15

**Authors:** Jacob M. Mallott, Eva M. Palacios, Jun Maruta, Jamshid Ghajar, Pratik Mukherjee

**Affiliations:** ^1^Department of Radiology and Biomedical Imaging, University of California, San Francisco, San Francisco, CA, United States; ^2^Departments of Neurology and Rehabilitation and Human Performance, Icahn School of Medicine at Mount Sinai, New York, NY, United States; ^3^Brain Trauma Foundation, New York, NY, United States; ^4^Department of Neurosurgery, Stanford University School of Medicine, Stanford, CA, United States; ^5^Department of Bioengineering and Therapeutic Sciences, University of California, San Francisco, San Francisco, CA, United States

**Keywords:** magnetic resonance, diffusion weighted imaging, acquired brain injury, fractional anisotropy, tract-based spatial statistics

## Abstract

Concussion, or mild traumatic brain injury (mTBI), is a major public health concern, linked with persistent post-concussive syndrome, and chronic traumatic encephalopathy. At present, standard clinical imaging fails to reliably detect traumatic axonal injury associated with concussion and post-concussive symptoms. Diffusion tensor imaging (DTI) is an MR imaging technique that is sensitive to changes in white matter microstructure. Prior studies using DTI did not jointly investigate white matter microstructure in athletes, a population at high risk for concussive and subconcussive head traumas, with those in typical emergency room (ER) patients. In this study, we determine DTI scalar metrics in both ER patients and scholastic athletes who suffered concussions and compared them to those in age-matched healthy controls. In the early subacute post-concussion period, athletes demonstrated an elevated rate of regional decreases in axial diffusivity (AD) compared to controls. These regional decreases of AD were especially pronounced in the cerebellar peduncles, and were more frequent in athletes compared to the ER patient sample. The group differences may indicate differences in the mechanisms of the concussive impacts as well as possible compound effects of cumulative subconcussive impacts in athletes. The prevalence of white matter abnormality in cerebellar tracts lends credence to the hypothesis that post-concussive symptoms are caused by shearing of axons within an attention network mediated by the cerebellum, and warrant further study of the correlation between cerebellar DTI findings and clinical, neurocognitive, oculomotor, and vestibular outcomes in mTBI patients.

## Introduction

Over 1.7 million Americans suffer a traumatic brain injury (TBI) each year (1). Moderate to severe TBI can often be diagnosed early through computed tomography (CT) and conventional magnetic resonance (MR) imaging. Concussion, or mild TBI (mTBI), however, makes up the great majority of TBI, but cannot be reliably detected by CT or conventional MR imaging techniques (2), which remain the standard of care.

The severity of a concussive injury is assessed by clinical evaluation of symptoms (3). Many concussed patients have symptoms including headaches, fatigue, insomnia, depression, attention problems, and memory problems (3), and while the majority recover within a few weeks, nearly a third continue to have persistent post-concussive symptoms (4–6). Current assessment does not reliably predict which mTBI patients will go on to suffer post-concussive symptoms (7); therefore, objective quantification of concussive injuries is needed.

Diffusion weighted imaging methods, including diffusion tensor imaging (DTI), provide useful tools for probing microstructural white matter changes in mTBI (2, 8). Traumatic axonal injury (TAI) may be inferred from CT or conventional MRI due to its association with small hemorrhages that those modalities can detect, but DTI can more directly detect changes in white matter microarchitecture (2). Past studies have found changes in DTI scalar metrics such as fractional anisotropy (FA), radial diffusivity (RD), and axial diffusivity (AD) in both acute and chronic mTBI patients, indicating microstructural alterations to white matter even in cases where CT and MRI scans were negative (7, 10). In the acute phase, FA has been seen to increase overall, while RD and AD have been seen to decrease. At chronic time points, the opposite effects are seen, with decreases in FA and increases in RD and AD (2, 9). Abnormal DTI scalar parameter values have been associated with cognitive functioning in mTBI patients (2, 8, 10, 11).

Prior DTI investigations of white matter microstructure have generally not studied different populations with concussions. However, athletes make up a population at high risk for concussive and subconcussive head traumas whose physical manifestations of injuries may differ from those due to mechanisms typically seen in the emergency room, such as motor vehicle collisions, assaults, and falls. The present study characterizes the early subacute abnormalities in DTI metrics in both athletes and ER patients after concussion, and compares the findings. In addition to the voxel-wise and tract-wise group comparisons of the conventional DTI literature, we also apply individual-patient tract-level analysis for greater clinical relevance and better robustness to the spatial heterogeneity of the white matter abnormalities associated with concussion (2, 7).

## Methods

### Subject Recruitment

This study was carried out in accordance with the recommendations of the institutional review board of the Weill Cornell Medical College (WCMC). All subjects gave written informed consent in accordance with the Declaration of Helsinki except, in the case of minors, legal guardians gave written informed consent with the assent of the subjects. The protocol was approved by the WCMC institutional review board, and data were collected from September of 2012 through September of 2016.

A concussion was defined as an event of blunt impact on the head with loss of consciousness (LOC), post-traumatic amnesia (PTA), or at least one of the following symptoms: dizziness, nausea, headaches, balance problems, blurred or double vision, or feeling dazed/confused. Although for the purpose of this research we did not rely on formal medical diagnosis of concussion necessary for clinical management of the injury (12), this definition is consistent with the guidance of the American Academy of Neurology (13). Eighteen scholastic athletes between ages 13 and 22 years were recruited for testing within 2 weeks of a concussion, as were 42 ER patients with concussion aged 7 years and older, of whom 18 were selected to match the age range of the athlete subjects. For the purpose of equity, athletes were enrolled independently of the level of contact involved in their participating sports. The recruited athlete and ER subjects were scanned for MR imaging as soon as possible. In addition, 38 control subjects aged 7 years and older with no prior history of head injury were recruited and received imaging, of whom 10 were selected to match the age range of the athlete subjects.

Subjects over 18 were required to have a high school diploma or GED; 18-year olds set to graduate high school on time were also included. Exclusion criteria for subjects were a prior history of eye disease, neurological/psychiatric conditions, or substance abuse (Table 1). Subjects with contraindications for an MRI were also excluded. For athletes and ER patients, additional exclusion criteria were acute intoxication at the time of the concussion and LOC or PTA for more than 24 h. Subjects' symptoms and cognitive performance were assessed with an extensive battery of tests as reported elsewhere (14, 15).

**Table 1:** Exclusion criteria.

**Basis**	**Details**
Neurological diagnosis	Prior Diagnosis of one or more of the following: Stroke, multiple sclerosis, epilepsy, brain tumor/cancer, encephalitis, dementia, movement disorder, spontaneous nystagmus
Eye-sight abnormalities	Amblyopia, uncorrected myopia, uncorrected presbyopia, uncorrected farsightedness, astigmatism, color blindness, macular degeneration
Eye diseases	Cataracts, glaucoma, retinal disorder
Psychiatric history	Any of the following: history of psychiatric hospitalization, history of legal trouble for violence, use of psychotropic medication other than a stable dose of SSRI
Psychiatric diagnoses	Prior Diagnosis of one or more of the following: Bipolar disorder, eating disorder, substance abuse disorder, personality disorder, sleep disorder, depressive disorder, anxiety disorder, ADHD
Questionnaires	Pediatric Subjects: *T*-Score ≥ 70 on Conners 3 Inattention Index or Hyperactivity Index, or *T*-Score ≥ 65 on BAI-Y or BDI-Y. 18+ Subjects: *T*-Score ≥ 70 on CAARS ADHD Index, ≥27 on CES-D, ≥26 on BAI.
Alcohol/drug abuse	Any of the following: • Score ≥ 6 on alcohol consumption survey • Answering 3 of 7 yes on MINI for alcohol dependence • History of daily/almost-daily use of illicit or prescription drugs • Use of any illicit or prescription drugs in past week • Past hospitalization/rehab for drugs • Past loss of job or suspension/expulsion from school for drugs • Multiple alcohol- or drug-related citation or arrest
MRI contraindications	Metal in body, claustrophobia, possibility of pregnancy

### Magnetic Resonance Imaging

MR imaging was performed at WCMC on a 3T Siemens Trio scanner. In each imaging study, whole-brain diffusion imaging was performed using an echo-planar imaging sequence (TE = 85 ms, TR = 7,500 ms) with one *b* = 0 s/mm^2^ scan and *b* = 1,000 s/mm^2^ in 64 diffusion directions. Imaging was performed with 128 × 128 × 60 cubic voxels of 2 mm dimensions.

Image preprocessing was performed using tools within the Functional MRI of the Brain (FMRIB, Oxford University, Oxford, UK) Software Library (16–18). Correction for eddy currents and subject motion was performed and registered to the *b* = 0 s/mm^2^ volume using the FMRIB Linear Image Registration tool (19). Image volumes were checked for excessive subject movement between diffusion weighted images and were accepted if mean and median movement were <2 mm.

**Figure 1:**
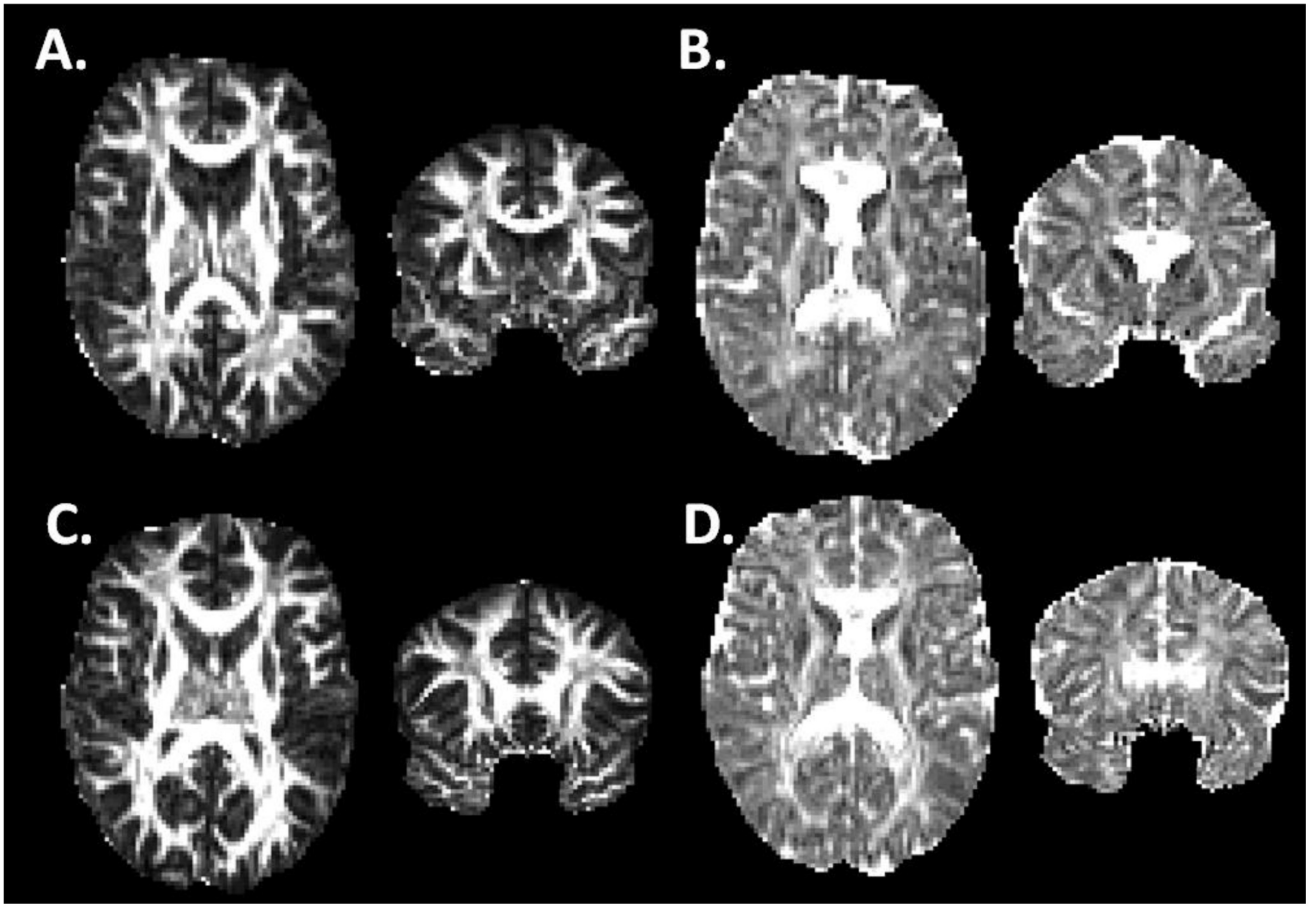
Fractional anisotropy and Axial Diffusivity in Athlete and Control Brains. **(A,B)** Fractional Anisotropy **(A)** and Axial Diffusivity **(B)** maps for one of the athlete subjects in horizontal and coronal views. **(C,D)** FA and AD maps for one of the control subjects in horizontal and coronal views. The imaging data has undergone image preprocessing, but has not yet undergone non-linear coregistration to a common template.

Non-brain voxels were then excluded using the FMRIB Brain Extraction tool (20). Using the diffusion-weighted data, a diffusion tensor model was generated using the FMRIB DTIFit algorithm, from which fractional anisotropy (FA), mean diffusivity (MD), radial diffusivity (RD), and axial diffusivity (AD) were determined at each voxel. Typical image quality is shown in Figure 1.

Tract-based Spatial Statistics (TBSS) were used to perform non-linear registration on the FA volumes to the FMRIB58_FA standard-space image, constructed from an average of 58 FA images taken of healthy subjects aged 20–50. After brain volumes were registered into a common space, a mean FA skeleton was generated using a threshold of FA ≥ 0.2 to limit the analysis to white matter voxels. TBSS alignment and white-matter skeleton generation was performed separately for each of the comparison groups (see *Statistical Analyses*) (21).

In addition to voxel-based analysis, masks were applied corresponding to the 27 white matter tracts previously labeled in the Johns Hopkins University (JHU) white-matter atlas (16), with bilateral tracts collapsed. Within each of these regions of interest (ROI), mean values for the four DTI scalar parameters were calculated for each subject.

### Statistical Analyses

Using the FMRIB Software Library randomize tool, permutation tests (*n* = 5,000) were performed to evaluate significant differences between groups on a voxel-wise basis, using Threshold-Free Cluster Enhancement, with correction for family-wise error. For each comparison group, permutation tests were performed for FA, MD, RD, and AD to control for false-positive voxels (22).

Mean FA, MD, RD, and AD within each of the 27 white-matter ROIs were compared between groups with two-sided *t*-tests, with false-detection rate (FDR) correction for the multiple comparisons. Comparisons were also made between the groups in terms of the number of individuals with extremely high or low parameter values for each of the four DTI metrics in each white matter tract. For this purpose, an abnormal parameter value was defined as >2.2 control group standard deviations above or below the control mean, which is the threshold used by Yuh et al. (7) in their DTI study of acute mTBI in ER patients. Significance of these group differences were determined with Pearson's χ^2^ test.

## Results

### Subject Demographics

Table 2 summarizes characteristics of the athlete and ER subjects. In our dataset, patient, and control ages were imperfectly matched, while differences in gender were not significant. Despite the best effort to have the recruited athlete and ER subjects scanned for MR imaging quickly, the timing of imaging since concussion ranged from 3 to 23 days, with an overall mean (SD) of 9.9 (4.1) days. There was no statistical difference in the timing of imaging between the athlete and ER groups.

**Table 2:** Subject demographics.

	**Athletes** **(18 subjects)**	**ER Patients** **(18 subjects)**	**Controls** **(10 subjects)**
Age	13–22 years (17.7 ± 3.0)	13–25 years (17.1 ± 3.7)	13–25 yrs (21.2 ± 4.0)
Gender	9 Female/9 Male	11 Female/7 Male	5 Female/5 Male
Time after injury	9.4 ± 5.2 days (3–23 days)	10.4 ± 2.7 days (4–14 days)	N/A
Inclusion criteria	All athlete subjects	Age-matched to athletes	Age-matched to athletes/ER patients

### Voxel-Wise Group Comparisons

**Figure 2:**
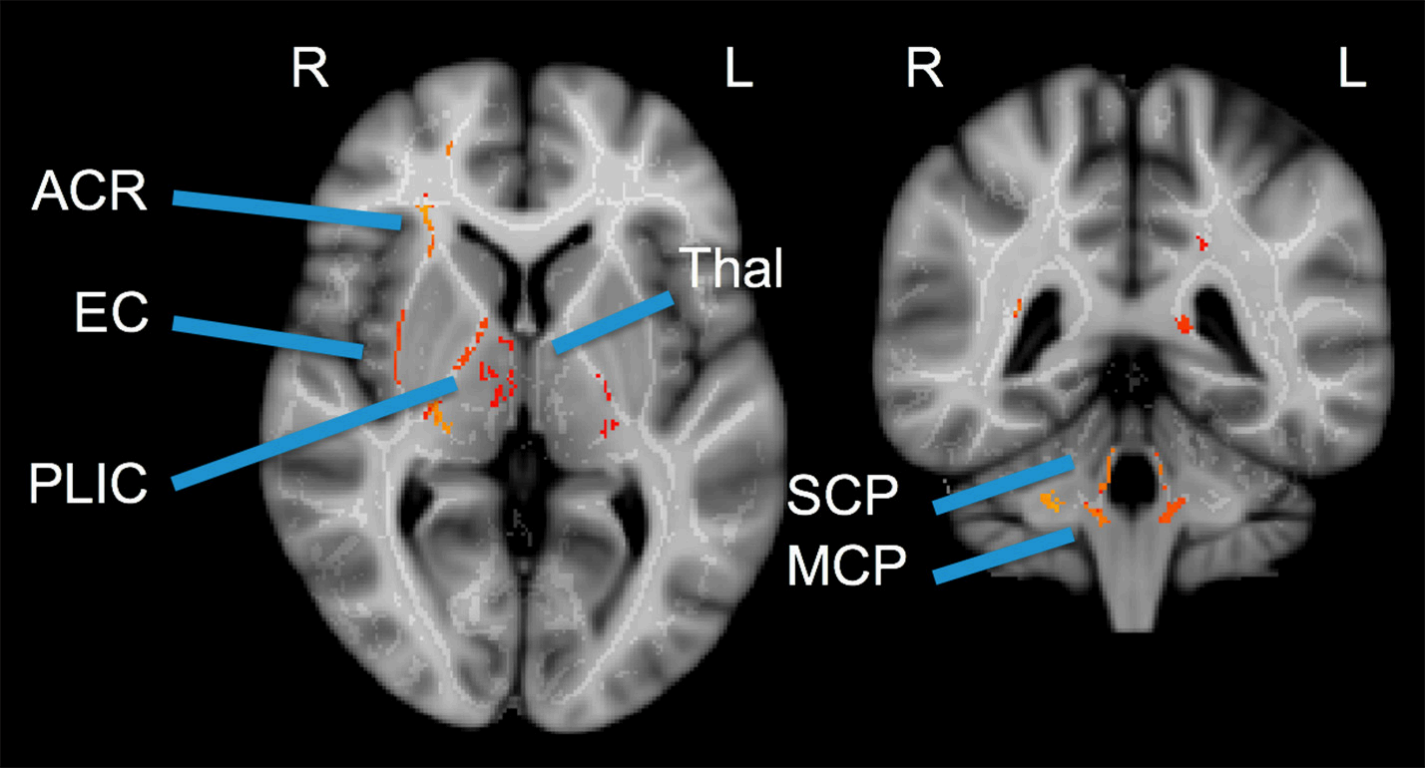
Cross-sectional voxel-wise comparison: Control > Patient Axial Diffusivity. Two cross-sections illustrating regions of significant decrease in athlete AD (acute post-injury time point) compared to controls. TBSS Analysis with Threshold-Free Cluster Enhancement FWE corrected at *p* < 0.025. ACR, Anterior Corona Radiata; EC, External Capsule; PLIC, Posterior Limb of Internal Capsule; Thal, Thalamus; SCP, Superior Cerebellar Peduncle; MCP, Medial Cerebellar Peduncle.

No significant differences between athlete and ER subjects were found in the voxel-wise comparison. For athletes, significant regional decreases were observed compared to controls in AD (Figure 2). These differences were seen in the uncinate fasciculus, external capsule, posterior limb of the internal capsule, and regions of the thalamus. All three of the cerebellar peduncles also saw significant decreases in AD.

### White Matter Tract Group Comparisons

Comparisons of white matter ROI means of DTI metrics demonstrated no significant group differences between athletes and controls or between ER patients and controls when adjusting for FDR (*q* = 0.2).

### Outlier/Abnormal Tract Analysis Using Normative Control Subjects

For each athlete and control subject, the number of high outlier and low outlier regions were counted for each DTI parameter (Table 3). Athletes had more abnormally low FA and AD, and high RD ROIs. The finding of abnormal regional low AD was the only one which remained significant after applying a Bonferroni correction. In athletes, the most common tracts with abnormally low AD were the middle cerebellar peduncle (11 athletes), inferior cerebellar peduncle (8 athletes), superior cerebellar peduncle (8 athletes), uncinate fasciculus (7 athletes), and the superior fronto-occipital fasciculus (5 athletes).

**Table 3:** Proportion of 18 athletes vs. 10 controls with one or more abnormally high/low DTI parameter value in a JHU atlas white matter tract.

	**Contains ≥ 1 JHU tract more than 2.2 SD above control mean**	**Contains ≥ 1 JHU tract more than 2.2 SD below control mean**
Fractional anisotropy	Control: 2 (20%) Athlete: 6 (33%) (*p* = 0.47)	**Control: 1 (10%)** **Athlete: 11 (61%) (*p* = 0.007)**
Mean diffusivity	Control: 1 (10%) Athlete: 6 (33%) (*p* = 0.19)	Control: 2 (20%) Athlete: 10 (56%) (*p* = 0.07)
Radial diffusivity	**Control: 0 (0%)** **Athlete: 8 (44%) (*p* = 0.011)**	Control: 1 (10%) Athlete: 7 (39%) (*p* = 0.11)
Axial diffusivity	Control: 1 (10%) Athlete: 5 (28%) (*p* = 0.29)	**Control: 2 (20%)** **Athlete: 16 (89%) (*p* < 0.001)**

*P-values determined by Pearson's χ^2^ test. Significant values in bold*.

The comparison of white matter ROIs in ER patients and controls showed similar trends, with significantly more ER patients demonstrating abnormally low FA and low AD in one or more ROIs compared to controls (Table 4). In ER patients, the most common tracts with abnormally low FA were the medial lemniscus (6 patients), posterior thalamic radiation (4 patients), and retrolenticular part of the internal capsule (4 patients). In ER patients, the most common tracts with abnormally low AD were the inferior cerebellar peduncle (6 patients), middle cerebellar peduncle (5 patients), and the uncinate fasciculus (4 patients).

**Table 4:** Proportion of 18 ER patients vs. 10 controls with one or more abnormally high/low DTI parameter value in a JHU atlas white matter tract.

	**Contains ≥ 1 JHU tract more than 2.2 SD above control mean**	**Contains ≥ 1 JHU tract more than 2.2 SD below control mean**
Fractional anisotropy	Control: 2 (20%) ER patient: 8 (44%) (*p* = 0.21)	**Control: 0 (0%)** **ER patient: 11 (61%) (*p* < 0.001)**
Mean diffusivity	**Control: 0 (0%)** **ER patient: 6 (33%) (*p* = 0.04)**	Control: 2 (20%) ER patient: 7 (39%) (*p* = 0.32)
Radial diffusivity	**Control: 0 (0%)** **ER patient: 9 (50%) (*p* = 0.005)**	Control: 1 (10%) ER patient: 5 (28%) (*p* = 0.29)
Axial diffusivity	Control: 1 (10%) ER patient: 8 (44%) (*p* = 0.065)	**Control: 2 (20%)** **ER patient: 11 (61%) (*p* = 0.038)**

*P-values determined by Pearson's χ^2^ test. Significant values in bold*.

### Athletes vs. ER Patients

Given the above results, we also compared the 18 athletes against the 18 ER patients. For the outlier analysis, we treated the ER patients as “controls” for determining means and standard deviations in ROI.

No significant differences were seen between the patient groups in voxel-wise or tract-wise group comparisons. However, the outlier analysis at the individual-subject level showed a significant number of athletes with one or more tracts exhibiting extremely low AD compared to the group of ER patients (Table 5). The athletes had the most low-AD counts in the fornix/stria terminalis (4 athletes), middle cerebellar peduncle (4 athletes), and the superior cerebellar peduncle (3 athletes).

**Table 5:** Proportion of 18 athletes vs. 18 ER patients with one or more abnormally high/low DTI parameter value in a JHU atlas white matter tract.

	**Contains ≥ 1 JHU tract more than 2.2 SD above ER mean**	**Contains ≥ 1 JHU tract more than 2.2 SD below ER mean**
Fractional anisotropy	Athletes: 7 (39%) ER patients: 4 (22%) (*p* = 0.29)	Athletes: 7 (39%) ER patients: 3 (17%) (*p* = 0.15)
Mean diffusivity	Athletes: 4 (22%) ER patients: 4 (22%) (*p* = 1)	Athletes: 5 (28%) ER patients: 2 (11%) (*p* = 0.22)
Radial diffusivity	Athletes: 5 (28%) ER patients: 3 (17%) (*p* = 0.44)	Athletes: 6 (33%) ER patients: 3 (17%) (*p* = 0.26)
Axial diffusivity	Athletes: 3 (17%) ER patients: 4 (22%) (*p* = 0.68)	**Athletes: 9 (50%)** **ER patients: 3 (17%) (*p* = 0.034)**

*P-values determined by Pearson's χ^2^ test. Significant values in bold*.

## Discussion

The present study shows early subacute post-concussion abnormalities in regional white matter microstructures in both scholastic athletes and ER patients. The abnormalities were observed as a greater frequency of extreme deviations in regionally defined DTI parameters, especially in the cerebellar peduncles of scholastic athletes, with our ROI-based outlier analysis. This method of analysis was modeled on that of (11) for chronic mTBI and Yuh et al. (7) for acute mTBI, in which high and low outliers in FA were compared between controls and patient subsets (7, 11). This method of classifying individual patients as having or lacking abnormal tracts can account for the spatial heterogeneity of mTBI, which often confounds group comparisons at both the voxel and tract spatial scales. This patient-specific analysis is also much more clinically relevant than findings at the group level.

Of particular note are our results demonstrating abnormally low AD both in the athlete and ER cohorts. The large majority of athletes had at least one white matter tract with a markedly low AD value, and while these low values were spatially heterogeneous, there was a striking consistency of having them in at least one of the three cerebellar peduncles. Some ER patients showed abnormally low AD values as well. Our direct comparison of ER patients and athletes suggests that those with sports concussions may be more prone to abnormalities in the cerebellar peduncles than typical ER concussion patients. The group differences may indicate differences in the mechanisms of the concussive impacts as well as possible compound effects of cumulative subconcussive impacts in athletes.

Our results are consistent with a prior DTI study showing decreases of white matter AD in male scholastic American football players at both 24 h and 8 days after concussion (23); however, that investigation focused on cerebral tracts and did not specifically investigate the cerebellum. A recent meta-analysis has shown variable findings from various DTI studies of sports concussions that may relate to differences in techniques and in the timing of imaging relative to the injury (24). Despite such differences, our study substantiates previous reports that suggest selective effects of TBI on the cerebellar peduncles—cerebellar white matter volume was found be reduced in children years after a TBI, implicating lasting cognitive and behavioral consequences (25), and FA alteration in cerebellum-related white matter tracts and associated cognitive deficits were found in acute-phase adult mTBI patients (26) and chronic phase combat veterans exposed to mTBI (27).

Neuropathological studies have shown cerebellar Purkinje cell loss in boxers (28) as well as in mice, non-human primates and combat veterans exposed to blast injuries (29, 30). The anatomic arrangement of the cerebellar peduncles as relatively unsupported bridges of white matter between the bulk of the cerebellum and the brainstem renders them uniquely vulnerable to shear stress from acceleration/deceleration effects, as shown in biomechanical studies (31–35).

These findings in the cerebellar peduncles provide some support to the hypothesis of predictive brain state disruption in TBI (36). By this hypothesis, the collection of clinically observed post-concussive symptoms could be explained by timing-related disruptions in an attention network mediated by the cerebellum (36), which was supported by a study that combined DTI, neurocognitive tests, and eye movement measurements (37). It should be noted that our analysis was data-driven across all of the major white matter tracts of the cerebrum and cerebellum; therefore, it was not influenced by any hypothesis regarding the location of injury such as the cerebellar peduncles. To further test the predictive brain state hypothesis, future studies can and should attempt to correlate cerebellar DTI findings to clinical, neurocognitive, oculomotor, and vestibular outcomes relevant to cerebellar function. This can pave the way toward targeted rehabilitation strategies aimed at improving the affected domains, especially attentional function which is one of the most disabling impairments in activities of daily living and can be ameliorated using goal-oriented mindfulness training regimens (38, 39).

A concern specific to our dataset is the wide window of “early subacute” post-concussion scan latencies. The differences between DTI parameters 3 days and 23 days after a concussion could be significant (9). The ROI-based outlier analysis proved to be a useful workaround for the range and variability in the timing of MR scan. Another limitation of the present study is that age matching between patients and controls was imperfect. On the whole, the control cohort was older than the concussion cohorts, as the total pool of control subjects was small. Since there are non-linear age-dependent changes in DTI measures that are also region-dependent, controlling for age by statistical regression is difficult (40). A linear regression, even done on a tract-by-tract basis, would not add any clarity to the results. Therefore, we did not attempt to use regression to adjust for age differences. However, the average age difference between the controls and mTBI patients is not expected to produce meaningful differences in the DTI measures, especially for most of the white matter tracts shown here to be significantly different between mTBI subjects and controls. Of special note, cerebellar white matter consists of early-maturing tracts (41) that reach their asymptotic DTI metric values during the first decade of life, with no significant changes in FA or MD during the rest of childhood or adolescence (42, 43). In particular, DTI metrics of all three cerebellar peduncles bilaterally plateau at 70 months of age (44). Therefore, no age-related changes in the cerebellar peduncles would be expected in the range of 13–25 years of the subject groups that we studied. Instead, any age-related differences between groups would have been observed in slower-maturing cerebral white matter, such as that of the frontal lobes. Finally, overall sample size was small. A larger statistical power may reveal more subtle group differences.

We did not exclude patients based on lesions that would be visible on routine clinical MRI. However, these otherwise healthy young athletes suffered concussions that rarely produce visible brain lesions, especially not in the cerebellum. Also, as our results are not explained by one or two outliers, we believe our results cannot be explained by lesions that could be detected by routine clinical MRI.

In designing future studies of athlete populations, it would perhaps be best to design image acquisition protocols that allow for more advanced diffusion models than DTI. For instance, neurite orientation dispersion and density imaging (NODDI) uses high angular resolution diffusion data with multiple *b*-values, and applies a three-compartment model that takes into account the neural microstructure in describing the diffusion pattern within a voxel (45). The NODDI model rests on certain assumptions regarding normal brain physiology, so in studying pathology such as mTBI, it is possible that the resulting NODDI parameters do not accurately describe the underlying brain architecture. However, Palacios et al have recently shown greater sensitivity in mTBI when studying changes in NODDI parameters compared to the DTI parameters analyzed in this study (46).

## Ethics Statement

This study was carried out in accordance with the recommendations of the institutional review board of the Weill Cornell Medical College (WCMC). All subjects gave written informed consent in accordance with the Declaration of Helsinki except, in the case of minors, legal guardians gave written informed consent with the assent of the subjects. The protocol was approved by the WCMC institutional review board.

## Author Contributions

JM, JG, and PM: conception and design. JM and JG: acquisition. JMM, EP, and PM: analysis. JMM, EP, JM, JG, and PM: interpretation.

### Conflict of Interest Statement

JG is director of Sync-Think, Inc., and the inventor of U.S. patent 7,384,399. JM holds stock option in Sync-Think. JG and JM are inventors of pending patent applications PCT/US2014/050774, PCT/US2016/027923, and US15585057 potentially related to the subject matter described in this article. PM declares research support from GE Healthcare as well as service on the Medical Advisory Board of the GE-NFL Head Health Initiative. The remaining authors declare that the research was conducted in the absence of any commercial or financial relationships that could be construed as a potential conflict of interest.
